# Perceived discrimination in medical settings and perceived quality of care: A population-based study in Chicago

**DOI:** 10.1371/journal.pone.0215976

**Published:** 2019-04-25

**Authors:** Maureen R. Benjamins, Megan Middleton

**Affiliations:** 1 Sinai Urban Health Institute, Sinai Health System, Chicago, Illinois, United States of America; 2 Chicago Medical School, Rosalind Franklin University of Medicine and Science, North Chicago, Illinois, United States of America; Indiana University, UNITED STATES

## Abstract

Perceived discrimination in medical settings remains prevalent within the U.S. health care system. However, the details of these experiences and their associations with perceived quality of care are not well understood. Our study assessed multiple measures of perceived racial/ethnic discrimination in medical settings and investigated the locations and purported perpetrators of the discriminatory experiences within a population-based sample of 1,543 Black, White, Mexican, Puerto Rican, and Other adults. We used logistic regression to estimate associations between perceived discrimination in the medical setting and three quality of care indicators. Overall, 40% of the sample reported one or more types of perceived discrimination in a medical setting, with significant differences by race/ethnicity. Discrimination was perceived across health settings and from a variety of providers and staff. In adjusted logistic regression models, individuals reporting discrimination had more than twice the odds of reporting fair or poor quality of care (OR = 2.4 [95% CI: 1.4–4.3]). In addition, perceived discrimination in medical settings was significantly associated with report of not having enough time with the physician and not being as involved in decision-making as desired. These findings expand our understanding of perceived discriminatory experiences in health care and the consequences of it for patients, providers, and health care systems. This information is essential for identifying future provider interventions and improving the training of health care professionals.

## Introduction

Racial and ethnic disparities in access to, and quality of, health care are pervasive and contribute to the persistent negative health outcomes seen among communities of color [1–7]. Perceived discrimination may underlie both disparities in health care and health outcomes [8,9]. In particular, research is needed to better understand racial and ethnic discrimination in the health care setting, which likely impacts health care perceptions and outcomes [10–13].

Patient perceptions of quality are important to study because there is a growing literature linking them with health care outcomes [14–15]. Perceptions of quality are now being incorporated into value-based incentive payments from the government and other organizations, as well as health care provider ratings [16]. Health care providers also rely on patient reports of quality to guide continuous quality improvement efforts. Given the increasing significance of perceived quality of care, more research is needed to understand the factors associated with it.

Despite this, only a handful of studies have examined the association between discrimination in health care and quality of care ratings, with most confirming the expected negative relationship [17–20]. More broadly, previous studies have found that perceiving discrimination in a medical setting is associated with other health care outcomes, such as delayed or unmet health care needs [20–23], underutilization of mental health services [24], and increased emergency department visits and hospital admissions [12]. However, the literature on discrimination in medical settings has been limited by measurement issues and unrepresentative samples [13]. For example, previous studies have commonly used a single question to assess whether or not an individual has perceived discrimination while receiving health care [10]. In addition, the majority of studies in this area focus on African American populations, with limited investigation into discrimination among other groups of color and between racial/ethnic populations typically combined into one group (e.g., Hispanics) [10, 13, 25].

Furthermore, there is a dearth of information about the purported perpetrators and settings of the discriminatory event [10]. One study, focusing on discrimination due to HIV status, assessed the perpetrators and found attributions spread among clinical staff (more than case managers or social workers) [26]. Another study examined the location of perceived discriminatory health care experiences and revealed that individuals using community clinics rather than doctor’s offices as their source of primary care reported higher rates of discrimination [27]. However, this finding was true only for the middle socioeconomic subgroup of participants.

To address these gaps in the literature, our study investigates the association of perceived discrimination in medical settings with three measures of perceived quality of care within a population-based sample from selected Chicago communities. Importantly, this sample includes sufficient data to examine four racial/ethnic groups (Non-Hispanic Black, Mexican, Puerto Rican, and Non-Hispanic White) and uses multiple measures of perceived discrimination, including a 7-item scale of discrimination in medical settings and questions that ask about discrimination due to insurance status and English proficiency. Finally, we also examine the purported perpetrators and settings of perceived discriminatory events. This new information can be used to inform the training of health care professionals and the development of policies within health care organizations to reduce perceived discrimination, with the goals of improved patient experience and greater health equity.

## Methods

### Sample

Our analysis used data from the Sinai Community Health Survey 2.0 (Sinai Survey 2.0). Detailed information on the sampling design and data collection methodology is available elsewhere (www.sinaisurvey.org). To summarize, the Sinai Survey 2.0 was administered by trained interviewers from the University of Illinois at Chicago (UIC) Survey Research Laboratory between March 2015 and September 2016. The sample was randomly selected from ten Chicago community areas. The communities were chosen based on geographic location and racial/ethnic composition, representing some of the most socially and economically challenged neighborhoods in Chicago. The sampled area represented approximately 386,000 individuals.

The community areas were divided into primary sampling units (PSU). The PSUs, and households within them, were randomly selected using Probability Proportionate to Size methodology. Selected households were mailed an advance letter with study information before the first contact. Following this, one or two adult(s) (18 years or older) were randomly selected from each household. An extensive protocol was followed to maximize the number of interviews completed, including up to ten in-person contact attempts, up to five telephone attempts, and materials left at the door.

Interviews were conducted face-to-face, in English or Spanish (depending on respondent preference) using computer-assisted personal interviewing software. The overall response rate, which includes some households with unknown eligibility, was 28.4% (calculated with American Association of Public Opinion Research’s (AAPOR) response rate number 3) [28]. The cooperation rate, or the proportion of respondents who completed an interview after contact, was 53.9% (AAPOR, response rate 4). The final sample included 1,543 adults. The Sinai Survey 2.0 was approved by the IRBs of the University of Illinois at Chicago (#2014–0524) and Mount Sinai Hospital (MSH #14–17). Interviewers explained the study to all participants, who then had the opportunity to ask questions before signing the informed consent document. The dataset and questionnaires are available through the Inter-university Consortium for Political and Social Research website (Sinai Community Health Survey 2.0, Chicago, Illinois, 2015–2016 (ICPSR 37073)).

### Measures

*Health Care Utilization*. Our dependent variable, perceived quality of care, was measured with the following question: “Overall, how would you rate the quality of health care you received in the last 12 months? Would you say excellent, very good, good, fair, or poor?” Those reporting fair or poor care were dichotomized from those reporting excellent, very good, or good care.

Two other, more specific, indicators of quality of care were also asked. These were slightly modified from the Commonwealth Fund Health Care Survey (2001) and reflect items from the Consumer Assessment of Healthcare Providers and Systems (CAHPS) survey related to clinician communication [29]. Both were prefaced with the instruction to think about one’s last visit to a doctor. The questions were: “How much did the doctor involve you in decisions about your care?” and “How much time did the doctor spend with you?” For both, respondents were given the following response options: “as much as you wanted, almost as much as you wanted, less than you wanted, or a lot less than you wanted.” The responses were dichotomized into positive and negative categories.

*Discrimination in Medical Settings*. For the primary independent variable, we used a slightly modified version of the Discrimination in Medical Settings scale (DMS) [12, 19, 30, 31], which is based on the Everyday Discrimination Scale (EDS) [32]. Questions were prefaced with the following: “Please think about all the times in your life when you’ve gotten health care. When getting health care, how often have any of the following things happened to you because of your race, ethnicity, or color?” During the questionnaire development, there was interest from several researchers in adding an item about providers acting as if they were unwilling to touch the patient, based on conversations with community members. Given the limited space available, the difficult decision was made to make room for this item by substituting it for another item from the scale. Because the item on being treated with less courtesy was perceived to overlap the most with the remaining items, it was excluded. The response scale for all items was never, rarely, sometimes, or often. Preliminary analyses showed that the responses had highly skewed distributions for each question, with 74–94% of responses falling in the “never” category. Thus, the responses were dichotomized to represent never experienced discrimination vs. ever (as done in previous uses of the scale) [12, 19].

For those reporting any lifetime experience of perceived discrimination in a medical setting (based on an affirmative answer to one or more of the DMS items), as well as a health care visit in the past 12 months, we asked about the individual involved and the location of the most recent time they perceived this type of discrimination. To identify the purported perpetrator, we asked, “Was the person who treated you this way your doctor, a nurse, a receptionist, or someone else?” Respondents could choose all that apply. The location measure assessed whether this most recent event took place in a doctor’s office, an emergency room, a clinic, or somewhere else. Respondents could only identify one location.

Other measures of perceived discrimination in medical settings included a question that assessed whether respondents felt that they had been “judged unfairly” or “treated with disrespect” by the doctor or medical staff due to their race, ethnicity, or color in the past 12 months using a four-point Likert-like scale ranging from 1 (never) to 4 (often). Perceived discrimination based on insurance-status and English proficiency was also assessed. Respondents were asked to think about their experiences in health care in the last 12 months and answer yes or no to the following questions: “Have you felt that the doctor or medical staff you saw judged you unfairly or treated you with disrespect because of the type or health insurance you have, or your ability to pay for care?” and “…because of how well you speak English?” These questions were only asked for those with a health care visit in the past 12 months.

*Demographic and Socioeconomic Characteristics*. Demographic variables included age, gender, and race/ethnicity (non-Hispanic White, non-Hispanic Black, Mexican, and Puerto Rican). Members of other racial/ethnic groups (including Hispanic and non-Hispanic individuals) were not examined in stratified analyses due to small sample sizes. Socioeconomic variables included education, employment, and current health insurance. Unmet health care needs measured whether or not respondents had health care needs in the past year that they did not receive due to cost. Health care needs included: medical care or surgery; a doctor’s appointment; prescription medicine; mental health care or counseling; dental care; and/or, eyeglasses.

*Other Covariates*. Global discrimination measured personal experiences of perceived discrimination due to race, ethnicity, or color using a four-point scale ranging from 1 (never) to 4 (often). These responses were dichotomized to reflect whether or not the participants had ever experienced discrimination. Self-rated health was assessed by asking: “Would you say that in general your health is excellent, very good, good, fair, or poor?” This variable was dichotomized into excellent, good, or very good health versus fair or poor health.

### Data analyses

We first generated descriptive statistics for the total sample and for the largest four racial/ethnic groups. Next, we examined bivariate relationships between our primary discrimination in medical settings outcome (DMS) and the three quality variables. Multivariate logistic regression models were also used to assess these associations, controlling for demographic, socioeconomic, unmet health care needs, and other covariates. For each of the three outcomes, both unadjusted and adjusted models were run. We also ran models with interaction terms between race/ethnicity and discrimination in health care to explore effect modification.

The survey team also conducted a nonresponse bias analysis to better understand potential bias due to the low response rate and sample selection (data not shown). Briefly, systematic block and housing unit observations were added to the data, along with block-group level data from the Census Bureau’s American Community Survey. Models were then run to identify variables that independently predicted the odds of 1) the primary respondent completing an interview and 2) having poor health outcomes or risk factors (as assessed by at least one negative response to 16 diverse health-related questions). Finally, any identified variables were used to re-estimate nonresponse-adjusted weights to account for potential nonresponse bias and estimates for each health indicator were compared with those using the previously estimated standard weights (which adjusted for gender, age, and race/ethnicity).

Statistical significance was set at p<0.05. All analyses were conducted in Stata 14.2 and account for the complex survey design. Estimates are also weighted to be representative of the entire population from which the sample was drawn.

## Results

### Descriptive statistics

#### Demographic and socioeconomic characteristics

Sample characteristics are shown in Table 1 for the full sample and by racial/ethnic group. The majority of the sample identified as a racial or ethnic minority. Survey respondents were evenly split between sexes and the mean age was 42 years. Approximately three-fourths of the sample had at least a high school degree and the vast majority were employed. Both Whites and Blacks had a significantly higher high school graduation rate than Mexicans, while Blacks were more likely to be unemployed than Whites or Puerto Ricans. Twenty-one percent of respondents were uninsured and one-third had unmet health care needs due to cost in the past year. White and Black adults were less likely to be uninsured than Hispanics; Blacks and Mexicans had higher rates of unmet health needs than Whites. Almost two-thirds of respondents had ever perceived discrimination in general. Whites were significantly less likely to report this than Blacks and Mexicans. Finally, one-third reported fair or poor health.

**Table 1:** Demographic, health, and health care characteristics by race/ethnicity^a^.

	Total	NH White	NH Black	Mexican	Puerto Rican
	Percent (95% CI)	Percent (95% CI)	Percent (95% CI)	Percent (95% CI)	Percent (95% CI)
***Demographics***					
Male	50 (46–54)	54 (43–65)	45 (40–50)	54 (49–60)	49 (38–59)
Age (mean)	42 (40–44)	47 (44–50)	42 (39–45)	39 (37–41)	45 (41–49)
English as primary language	86 (81–89)	100^b^	100^b^	70 (62–77)	89 (74–96)
Married	38 (33–42)	51 (39–63)	20 (15–27)	45 (38–52)	34 (23–46)
***Socioeconomic Status***					
High school degree or more	73 (68–78)	99 (97–100)	78 (71–84)	62 (52–70)	68 (51–81)
Unemployed	10 (8–12)	3 (1–8)	16 (11–21)	8 (5–12)	4 (2–9)
***Access to Care***					
Health insurance	79 (75–83)	93 (87–97)	89 (84–93)	66 (58–73)	66 (58–73)
Any unmet health care needs	33 (29–38)	19 (12–27)	42 (35–49)	32 (35–49)	33 (22–47)
***General Discrimination***					
Any discrimination	64 (59–69)	37 (26–48)	73 (66–79)	67 (60–74)	63 (47–76)
***Self-Rated Health***					
Fair or poor	33 (29–37)	18 (10–28)	34 (26–43)	37 (31–42)	31 (20–45)
***Health Care Quality Outcomes***					
Perceived quality of care as fair/poor	15 (12–19)	3 (1–6)	16 (12–22)	20 (14–28)	11 (5–25)
Not involved in decisions	7 (5–9)	8 (5–14)	6 (4–9)	8 (5–13)	5 (2–12)
Little time with doctor	15 (12–19)	10 (6–17)	15 (10–22)	17 (12–25)	18 (10–32)
***N***	1,543 ^c^	219	536	521	151

Notes: NH = non-Hispanic; CI = confidence interval

^a^ *Sinai Community Health Survey 2*.*0* (2015–2016, ten Community Areas in Chicago, IL). All statistics weighted for clustered survey sampling design.

^b^ No confidence interval.

^c^ Total sample includes individuals in racial/ethnic groups other than those displayed separately.

#### Health care outcomes

Fifteen percent reported receiving fair or poor quality health care in the past year. This number was significantly lower for White respondents compared to Blacks and Mexicans. Seven percent reported that they were involved in decisions less than they wanted and 15% had less time with the doctor than they wanted. These numbers did not vary significantly by race/ethnicity.

#### Discrimination in medical settings

Overall, 40% had ever experienced some type of discrimination in a medical setting (DMS) (Table 2). This differed significantly by race/ethnicity, with Whites being less likely to report discrimination than the three groups of color and Mexicans being less likely to report it than Blacks. The scale item “received poorer service” was most frequently reported (29%). Again, significant racial/ethnic differences exist. For example, only 3% of Whites reported experiencing this type of discriminatory event, compared to 43% of Blacks. The scale item “…doctor or nurse acts as if he or she is afraid of you” had the lowest overall affirmation rate (6%); however, among Blacks, 14% perceived this type of discrimination.

**Table 2:** Perceived discrimination in medical settings and related factors by race/ethnicity, *Sinai Community Health Survey 2*.*0* (2015–2016, ten Community Areas in Chicago, IL)^a^.

	Overall	NH White	NH Black	Mexican	Puerto Rican
	Percent (95% CI)	Percent (95% CI)	Percent (95% CI)	Percent (95% CI)	Percent (95% CI)
**Discrimination in Medical Settings (ever)**					
*Any Type of Discrimination in Medical Settings*	40 (35–45)	13 (8–20)	56 (48–63)	39 (32–46)	44 (31–58)
Doctor or nurse is not listening to you	24 (20–28)	4 (2–11)	34 (29–41)	23 (18–30)	28 (16–43)
Treated with less respect	25 (20–29)	3 (1–9)	34 (27–42)	25 (19–31)	29 (17–44)
Received poorer service	29 (24–34)	3 (1–10)	43 (35–51)	28 (22–34)	33 (21–48)
Doctor or nurse acts better than you	18 (15–22)	7 (4–11)	26 (20–32)	15 (10–20)	23 (13–39)
Doctor or nurse acts as if you are not smart	18 (14–22)	7 (3–13)	29 (23–36)	14 (10–19)	26 (14–42)
Doctor or nurse acts as if he or she is afraid of you	6 (4–9)	-- ^b^	14 (10–21)	4 (2–7)	5 (2–11)
Doctor or nurse did not want to touch you	8 (6–10)	-- ^b^	14 (11–19)	6 (4–10)	5 (2–11)
**Treated Unfairly When Getting Medical Care (past year)**^c^					
Never	82 (77–85)	100 (98–100)	80 (73–85)	76 (68–83)	85 (69–93)
Rarely	11 (8–15)	-- ^b^	10 (7–15)	17 (11–24)	8 (2–22)
Sometimes	6 (4–9)	-- ^b^	8 (5–14)	6 (3–11)	7 (2–23)
Often	1 (0–3)	-- ^b^	2 (1–4)	2 (1–4)	-- ^b^
**Purported Perpetrator of Most Recent Discriminatory Event**^d^^e^					
Doctor	25 (19–33)	29 (10–58)	30 (19–45)	22 (13–36)	16 (7–31)
Nurse	30 (24–36)	21 (7–48)	31 (23–41)	24 (17–34)	39 (19–63)
Receptionist	23 (17–29)	7 (3–19)	18 (10–30)	29 (22–37)	26 (11–51)
Someone else	22 (17–28)	-- ^b^	25 (18–34)	23 (16–33)	13 (6–27)
**Location of Most Recent Discriminatory Event**^e^					
Doctor’s office	29 (22–36)	19 (8–37)	33 (22–48)	25 (15–38)	34 (18–55)
Emergency room	30 (23–38)	44 (19–73)	32 (20–47)	31 (22–41)	28 (12–52)
A clinic	20 (15–25)	-- ^b^	19 (13–28)	18 (12–28)	17 (7–36)
Somewhere else	22 (17–28)	31 (12–60)	16 (10–24)	26 (18–37)	21 (7–50)
**Judged or Treated Unfairly by Doctor or Staff**^c^					
Because of type of insurance or ability to pay	13 (11–17)	7 (4–12)	22 (16–29)	10 (6–15)	15 (7–32)
Because of how well you speak English	2 (0–8)	-- ^b^	4 (2–7)	11 (7–15)	5 (2–13)
***N***	1,543	219	536	521	151

*Notes*: NH = non-Hispanic; CI = confidence interval

^a^ All statistics weighted for clustered survey sampling design.

^b^ Insufficient cell size (n<5).

^c^ Limited to those with a health care visit in past 12 months.

^d^ Respondents could choose more than one answer.

^e^ Limited to those with a health care visit in past 12 months and who reported a discriminatory event.

Overall, a smaller percentage reported discrimination in a medical setting when it was asked as a single question (separate from the DMS scale) and limited to experiences in the past year. For example, only 18% reported any level of perceived discrimination in a medical setting when asked in this manner. Affirmative responses ranged from no Whites reporting this to one-quarter of Mexicans. Very few responded that this type of discrimination occurred “often”.

*Purported Perpetrator*. Those who responded affirmatively to any of the DMS items and had a health care visit in the past year were asked to identify the perpetrator(s) of the most recent time they perceived being discriminated against. A substantial percentage selected each of the four potential options (i.e. doctor, nurse, receptionist, or someone else). The only racial/ethnic difference was that Whites were significantly less likely to report perceived discrimination from a receptionist compared to Mexicans.

*Location*. Similar to above, respondents who reported any type of perceived discrimination in the DMS and who had a health care visit in the past year were asked about the location of the most recent discriminatory event. Responses were evenly distributed between the four options of a doctor’s office, the emergency room, a clinic, and elsewhere. Whites were less likely to report such experiences in a clinic setting compared to the other groups, but no racial/ethnic differences were seen.

*Judged Unfairly*. Overall, 13% of those with a health care visit in the past year reported that they had been judged or treated unfairly by a doctor or staff member because of their type of insurance or ability to pay. Blacks were significantly more likely to report this than Whites and Mexicans. Overall, only 2% reported being treated unfairly due to how well they spoke English. Whites were significantly less likely to report this type of perceived discrimination compared to other groups.

### Bivariate analyses

**Fig 1:**
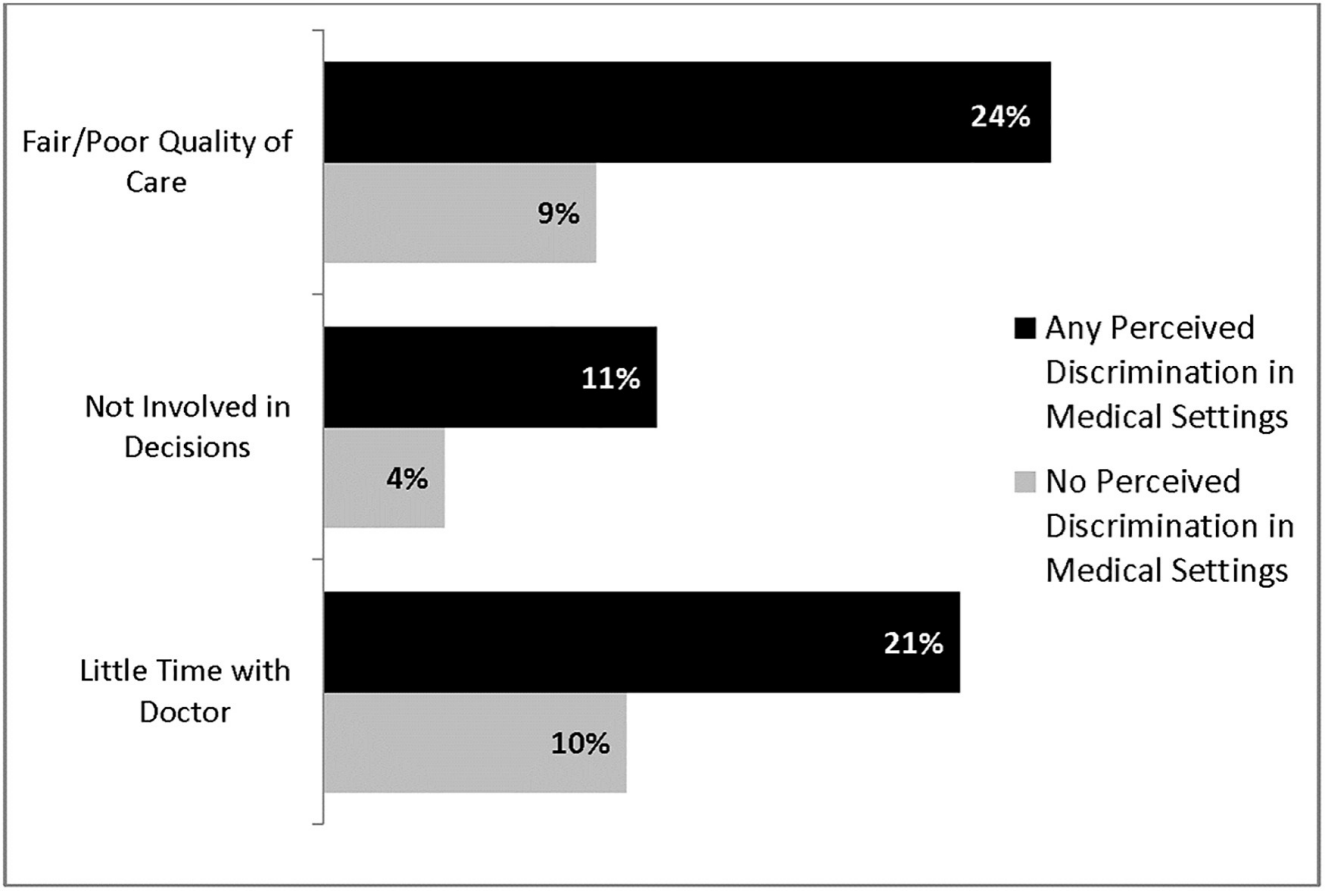
Three measures of health care quality by lifetime experience of perceived discrimination in medical settings^ab^. ^a^ *Sinai Community Health Survey 2*.*0* (2015–2016, ten Community Areas in Chicago, IL). All statistics weighted for clustered survey sampling design. ^b^ “Any Perceived Discrimination in Medical Settings” indicates an affirmative response to any item in the Discrimination in Medical Setting scale.

Fig 1 displays results from the bivariate analyses. Those reporting DMS were more than twice as likely to rate their quality of care as fair or poor compared to those with no DMS (24% [95% CI: 18–30%] versus 9% [95% CI: 6–13%], respectively). Those reporting DMS were also significantly more likely to be less involved in decisions than they wanted (11% [95% CI: 8–15%]) versus 4% [95% CI: 3–7%], respectively)) and have less time with the doctor than they wanted compared to those reporting no DMS (21% [95% CI: 17–27%]) versus 10% [95% CI:7–16%], respectively).

### Logistic regression analyses

We report unadjusted and adjusted odds ratios in Table 3. The findings from the six logistic regression models indicate that DMS is significantly associated with all three quality of care outcomes (i.e. overall perceptions of health care quality as fair or poor, less involvement in health care decisions than desired, and less time spent with one’s physician than desired). In the three unadjusted models, individuals who report any perceived discrimination in a medical setting have two to three times higher odds of reporting each of the negative health care quality outcomes.

**Table 3:** Unadjusted and adjusted odd ratios for the association of perceived discrimination in medical settings with three health care quality outcomes^a^.

	Perceived Quality of Care (Fair/Poor)	Not Involved in Decisions	Little Time with Doctor
	OR (95% CI)	OR (95% CI)	OR (95% CI)
**Discrimination in Medical Settings**			
Unadjusted Models	3.2 (1.9–5.3)	2.9 (1.6–5.2)	2.4 (1.4–4.2)
	*n = 1*,*397*	*n = 1*,*529*	*n = 1*,*530*
Adjusted Models ^b^	2.4 (1.4–4.3)	2.4 (1.3–4.4)	2.6 (1.5–4.6)
	*n = 1*,*243*	*n = 1*,*362*	*n = 1*,*364*

Notes:

^a^ *Sinai Community Health Survey 2*.*0* (2015–2016, ten Community Areas in Chicago, IL). All statistics weighted for clustered survey sampling design.

^b^ Adjusted for age, sex, race/ethnicity, marital status, education, employment, insurance, unmet health care needs, general discrimination, and self-rated health. Excludes members of Other race/ethnic category.

In the three adjusted models, two of the odds ratios are slightly attenuated, but all remain significant. Specifically, individuals reporting perceived discrimination in a medical setting had more than twice the odds of reporting fair or poor quality of care (OR = 2.4 [95% CI: 1.4–4.3]). Perceived discrimination in medical settings was also significantly associated with not being as involved in decision-making as desired (OR = 2.4 [95% CI: 1.3–4.4]) and not having enough time with the physician (OR = 2.6 [95% CI: 1.5–4.6]).

Results from models including the interaction terms between race/ethnicity and discrimination in health care (not shown) suggested that effect modification was likely for two of the three quality outcomes (perceived quality of care and involvement in decisions). In these models, discrimination in health care was most strongly associated with the quality outcomes for Blacks and Mexicans. However, the small sample sizes precluded stratifying models by race/ethnicity.

### Nonresponse bias

In the nonresponse bias analyses (not shown), two variables—concentrated economic disadvantage (percent unemployed, percent female-headed families with children, percent Black, and percent below the poverty level) and concentrated immigration (percent Hispanic and percent foreign-born)—were associated with both nonresponse and at least one of the 16 selected health measures. When comparing estimates for each health indicator using the standard weights and the nonresponse-adjusted weights, all estimates were similar (i.e. none of the nonresponse-adjusted health indicator estimates fell outside the 95% confidence intervals of the standard weighted estimates).

## Discussion

A positive patient experience is increasingly recognized as an essential element of high quality health care [33]. Accordingly, perceptions of quality of care are now included in determinations of governmental funding and provider ratings. Emerging literature has found that perceptions of low-quality care may be linked to poorer health care outcomes (such as delayed care and non-compliance with treatment) as well as objective measures of quality of care [14, 22]. However, little is known about the predictors of patient quality ratings. Our study adds to the evidence by exploring how frequently perceived discrimination in medical settings is reported, who is allegedly perpetrating it, where it happens, and how it is associated with perceived quality measures.

Overall, 40% of adults in this sample responded affirmatively to one or more items on the DMS, with significant differences by race/ethnicity. Receiving poorer service was the most frequently reported issue, with over 40% of Blacks reporting this type of discriminatory event. Few patterns were seen regarding the purported perpetrators and locations identified. More specifically, doctors, nurses, receptionists, and others were all sources of perceived discrimination. Similarly, substantial percentages of the population reported perceived discriminatory events at each type of clinical location. Finally, in adjusted models, reporting one or more types of discrimination in a medical setting was significantly associated with reporting fair or poor quality of care, not having enough time with the physician, and not being as involved in decision-making as desired.

### Comparison with previous findings

Previous studies using the DMS scale reflect a wide range of prevalence rates, from 8% of female veterans [34] to over 60% of those included in a convenience sample of African American adults in northern Ohio [12] and a sample of HIV patients [29]. Consistent with the current literature, we found that the perception of race-related fear from doctors or nurses toward patients was the least reported type of perceived discrimination [12, 30, 34, 35]. In previous studies, perceiving that providers were “not listening” was the most commonly reported item on the DMS [12, 19, 29, 30, 34]; however, in our study, receiving “poorer services” was most frequently reported.

Not surprisingly, our study found lower levels of perceived discrimination in health care (18%) when using a single-item measure and when limiting events to the past 12 months. Many previous cross-sectional studies on general populations (also using single-item question) have reported far lower rates of health care discrimination, ranging from 1% to 9%, compared to studies using the DMS [17, 22, 24, 36–44]. Most of these had predominantly White sample populations [22, 24, 36–39]. In contrast, a cross-sectional study conducted in Chicago in 2002 yielded a higher prevalence of 22% [20].

Consistent with much of the previous literature, our study found that Blacks reported the highest rates of health care discrimination (56%) using the DMS scale [10, 45, 39, 43, 44]; however, rates among Puerto Ricans and Mexicans far exceeded previous findings at 45% and 40%, respectively. Few studies have estimated prevalence rates within the large and diverse Latino population [17, 41]. One study of Puerto Ricans in Boston found that 12% had experienced discrimination in medical settings [46]. Another found nearly equal rates of perceived healthcare discrimination between adults of Mexican and Puerto Rican descent, at roughly 24% [20].

### Perceived discrimination and perceived quality of health care

The current study found that DMS is associated with three measures of quality of care (related to overall perceived quality, involvement with decisions, and time spent with physician), in line with the existing studies. For example, one study found that perceiving discrimination while receiving health care was the primary predictor of the variance in quality of care ratings between Black and White patients [17], while another found that perceived discrimination in health care (not necessarily due to race/ethnicity) was related to perceived quality of care for foreign-born (but not U.S. born) Hispanics [18]. Most existing studies on perceived discrimination in health care and quality outcomes have used single item discrimination measures [17, 18, 30, 43, 47]. As an exception, a study using a small sample of Black and White veterans found that a multi-item scale of discrimination in health care was associated with quality of diabetes care [19].

### Strengths and limitations

The present research builds upon the literature by examining the prevalence of this type of discrimination for adults of Mexican and Puerto Rican descent, which addresses recent calls to add to the limited body of health care discrimination research on these growing populations [25, 27, 41]. In addition, we were able to enlist the more detailed DMS tool in order to attain more actionable and specific data regarding perceived discriminatory experiences in health care settings. This study is also among the first to assess the locations and purported perpetrators of perceived discrimination in health care. It is critical to understand that the patient experience, and perceptions of quality, are impacted at all stages of the clinical process, from ease of scheduling to interactions with office staff (who are often a patient’s first point of contact), to physician encounters [48]. By studying the purported perpetrators and locations reported in the context of discriminatory events, we are able to more clearly understand the patient experience and, consequently, collectively work to improve patient care and health outcomes through targeted interventions.

It should be noted that these data, which were collected using a probability sample of ten diverse Chicago communities, do not represent greater Chicago or U.S. populations. As discussed above, the overall response rate was low (28.4%, AAPOR response rate type 3) [28]. We weighted all statistics to make the results representative of the sampling frame to account for this. Moreover, the cooperation rate, which only includes those individuals with whom interviewers made contact, was almost double (53.9%, AAPOR cooperation rate type 4) [28]. These rates reflect the general decline in response rates seen nationally. As with all surveys, we must consider whether participants differ from non-participants in characteristics related to our variables of interest (or the relationships between them). Our nonresponse bias analysis did not show evidence of this type of bias in these data. However, we were unable to specifically test for differences in levels of perceived discrimination or healthcare satisfaction between responders and nonresponders. One might hypothesize that individuals with higher levels of perceived discrimination in medical settings and lower levels of healthcare satisfaction would be less likely to participate than other individuals, particularly given that our survey was conducted by a research center that is part of a healthcare system. If this was the case, our numbers underestimate the extent of perceived discrimination in our healthcare system, while overestimating levels of satisfaction with care.

Another of our study’s weaknesses is intrinsic to the measure of perceived discrimination. Self-reported discrimination measures reflect only what subjects are able to identify and recall, and willing to report. Moreover, several measures related to discrimination in a medical setting were limited to those who reported a health care visit in the past year. The use of odds ratios as a measure of association may overstate the association, given that both the outcome and exposure are prevalent. Finally, the cross-sectional nature of the data precluded investigation of causal relationships.

### Practical implications

By illuminating associations between patients’ perceptions of discrimination in medical settings and quality of care outcomes, health care systems can be incentivized and guided in the development of systemic improvement measures. Such interventions, including surveillance, training, and policies, can be further informed by data regarding the types of behaviors and clinical scenarios most commonly implicated in perceived discriminatory experiences. Awareness of the need for this type of work is increasing, as highlighted by the Physicians’ Charter which states that physicians must “work actively to eliminate discrimination in health care” [49]. Though previous studies of the effectiveness of culturally competent care education have shown mixed results [50], there is some evidence that completion of such interventions can mediate greater patient satisfaction and greater follow-up appointment attendance [51]. Similarly, medical schools have shown some success in reducing medical student implicit bias through health disparities and culture competence curricula [52]. Strategies to specifically address physician implicit bias have been proposed [53], but there is minimal implementation of this type of training in practice [54].

### Directions for future research

Larger and national studies of perceived discrimination in medical settings are needed to assess the prevalence and consequences of this issue in the broader U.S. health care system [10]. Future studies, following patient perceptions, health behaviors, and outcomes over time, would help clarify which health care outcomes are most vulnerable to this type of perceived discrimination. Learning health care systems working to reduce provider-based discrimination through professional training (such as implicit bias training) should carefully evaluate and report on the efficacy of such systemic implementations. Ideally, objective health outcomes from EMR data should be assessed to better understand the impact of health care discrimination perceptions on health, as well as potential improvements resulting from system interventions. Ultimately, however, we must recognize that the factors contributing to health disparities are vast, and extend far beyond the direct interactions between patients and health professionals [2]. Further research into the influence of institutional racism and other social determinants of health is needed to guide broader policy-making that might work synergistically with improvements in interpersonal health care experiences, with the ultimate goal of improving health equity [10, 55].

## Conclusions

Levels of perceived discrimination in medical settings over a lifetime are high for Blacks, Mexicans, and Puerto Ricans in this sample from ten Chicago communities and this type of perceived discrimination was strongly related to perceptions of the patient experience. While racism is widely acknowledged (and considered a determinant of existing health disparities) for Black populations, perceived discrimination *in the medical setting* represents an emerging issue to be addressed for the growing Hispanic populations and those who serve them. Our findings further reveal that perceived discriminatory events are not limited to one type of health care setting or role. More work is needed to identify, address, and, hopefully, prevent, this type of interaction within our health care systems in order to improve patient experiences and ultimately outcomes.
